# Impacts of pristine, aged and leachate of conventional and biodegradable plastics on plant growth and soil organic carbon

**DOI:** 10.1007/s11356-024-31838-9

**Published:** 2024-01-15

**Authors:** Amy C. M. Wright, Bas Boots, Thomas C. Ings, Dannielle S. Green

**Affiliations:** Applied Ecology Research Group, School of Life Sciences, Anglia Ruskin University, Cambridge, CB1 1PT UK

**Keywords:** Biodegradable plastics, Micro- and meso-plastics, Aged plastics, Leachate, Soils

## Abstract

Plastic is an essential component of agriculture globally, becoming a concerning form of pollution. Biodegradable alternatives are gaining attention as a potential replacement for commonly used, non-degradable plastics, but there is little known about the impacts of biodegradable plastics as they age and potential leachates are released. In this study, different types (conventional: polyethylene and polypropylene and biodegradable: polyhydroxybutyrate and polylactic acid) of micro- and meso-films were added to soil at 0.1% (w/w) prior to being planted with *Lolium perenne* (perennial ryegrass) to evaluate the plant and soil biophysical responses in a pot experiment. Root and shoot biomass and chlorophyll content were reduced when soil was exposed to plastics, whether conventional or biodegradable, pristine, aged or when just their leachate was present. The pH and organic matter content of soil exposed to these plastics and their leachates was significantly reduced compared to control samples; furthermore, there was an increase in CO_2_ respiration rate from soil. In general, meso (> 5 mm) and micro (< 5 mm) plastic films did not differ in the impact on plants or soil. This study provides evidence that conventional and biodegradable plastics have both physical and chemical impacts on essential soil characteristics and the growth of *L. perenne*, potentially leading to wider effects on soil carbon cycling.

**Graphical abstract:**

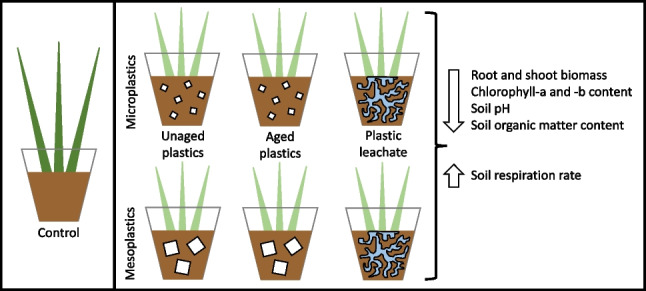

**Supplementary Information:**

The online version contains supplementary material available at 10.1007/s11356-024-31838-9.

## Introduction

Plastic pollution, recognised by the United Nations Environment Program (UNEP) as a major threat to soil health and global food security (FAO and UNEP 2021), has raised growing concerns, with research by the Food and Agriculture Organisation of the UN (FAO) and UNEP indicating that agricultural soils may receive higher volumes of microplastics (particles < 5 mm in size (Frias and Nash 2019)) than oceans (FAO and UNEP 2021). Globally, the use of plastic in agriculture is increasing. The FAO (2021) estimates that the current annual global consumption of plastic within the agricultural industry is 12.6 million tonnes, making agricultural soil particularly prone to microplastic contamination. There are several pathways for plastic contamination into agroecosystems, including from fertiliser in the form of sewage sludge (Corradini et al. 2019), atmospheric deposition and precipitation (Dris et al. 2016; Bergmann et al. 2019), and plastic agricultural equipment, particularly the use of mulching sheets (Blasing and Amelung 2018; Huang et al. 2020; Kumar et al. 2020; Lozano et al. 2020; Wang et al. 2022). The use of plastic in mulch films has become a fundamental part of intensive agriculture (Ekebafe et al. 2011). The FAO (2021) states that the agricultural plastic industry predicts that the current global plastic film demand of 6.1 million tonnes (2018) will rise by 50% to 9.5 million tonnes in 2030. Mulch films can improve crop productivity and yield by regulating soil temperatures, retaining soil moisture, preventing soil erosion and subsequently nutrient loss and reducing the need for fertiliser, pesticide and herbicide use by inhibiting weed growth and minimising contact with pests and diseases (Kasirajan and Ngouajio 2012; Viljoen et al. 2023). According to Zhou et al. (2023), the lifespan of these films, however, is short (< 1 year for outdoor mulches and ca. 5 years when used within greenhouses), due to a series of aging mechanisms, such as light (UV) degradation, wind and water erosion and microbial decomposition (Wang et al. 2021). The breakdown of mulch films potentially causes the accumulation of smaller plastic pieces such as microplastics and meso-plastics (Liu et al. 2018). Polyethylene (PE) and polypropylene (PP) are the most frequently found polymers in soil globally, corresponding to commonly used mulch film polymers (Kasirajan and Ngouajio 2012; Huang et al. 2020).

Biodegradable plastics have gained significant attention as a potential replacement for non-degradable, conventional plastics (Qin et al. 2021). For example, biodegradable plastic mulch films have been developed to replace those made from conventional plastics (PE and PP) (Bandopadhyay et al. 2018). Common polymers used in these biodegradable mulches include polylactic acid (PLA) and polyhydroxyalkanoates (PHA), such as polyhydroxybutyrate (PHB) (Kasirajan and Ngouajio 2012). Biodegradable polymers are, in theory, susceptible to microbial hydrolysis (Brodhagen et al. 2015), meaning that soil microorganisms can completely metabolise these bioplastics into microbial biomass, CO_2_ and water (Lucas et al. 2008; Bano et al. 2017; Luyt and Malik 2019). This degradation is controlled by environmental factors such as temperature, moisture levels and the presence of plastic-degrading microorganisms (Brodhagen et al. 2015); however, it is possible that plastic-degrading bacteria do not always respond to bioplastics and, even when biodegraded, additive residues may remain in the soil (Goel et al., 2021). In practice, research suggests that full degradation of biodegradable materials is often not achieved in the environment under natural conditions (Kubowicz and Booth 2017; Viera et al. 2020).

Despite the increasing use of biodegradable mulching films, preliminary research shows that, when compared to conventional polymers, biodegradable plastics may have equal impacts to the following: the germination and growth of ryegrass (*Lolium perenne*) (Boots et al. 2019), common bean (*Phaseolus vulgaris* L.) (Meng et al. 2021) and wheat (*Triticum aestivum*) (Qi et al. 2018, 2020b); soil physicochemical properties including structure, bulk density, porosity, and water holding capacity (Qi et al. 2020a); and soil invertebrates, such as earthworms (*Aporrectodea rosea* and *Eisenia fetida*) (Boots et al. 2019; Ding et al. 2021). Different plastics have elicited differing responses in both soils and plants, likely due to their different compositions (including chemical additives added to the polymer), sizes, differing deposition rates (Xu et al. 2020; Liu et al. 2023a) and differing degradation rates (bioplastics may break down into microplastics faster than conventional plastics (Brodhagen et al. 2015)). For example, Boots et al. (2019) found PLA exposure (0.1% w/w) to decrease the germination of *L. perenne* seeds by 6% decrease, while there was no significant change observed with HDPE (Boots et al. 2019). Boots et al. (2019) also report a decrease in soil pH with exposure to (high density) PE microplastics (0.1% w/w), hypothesising that this change could be due to microplastic particles altering soil cation exchange capacity by enabling the free exchange of protons in the soil water, resultant from their large surface area. In contrast, Zhao et al. (2021) found conventional microplastic films (0.4% w/w) to increase soil pH, potentially due to increases in soil aeration and porosity (Lozano et al. 2021). An altered soil pH can affect soil microbial growth and metabolism (Bahram et al. 2018; Crowther et al. 2019), which could be detrimental to ecological processes such as nutrient cycling and soil organic matter decomposition (Yan et al. 2017a; Kang et al. 2021).

Despite plastic mulching being widely used in agriculture, and thus leading to the accumulation of microplastic films in soil (Steinmetz et al. 2016), there exists a strong research focus on fibrous microplastics, with microfilms being largely neglected (Lehmann et al. 2021; Zhao et al. 2021). Several studies have identified the size distribution of agricultural plastic deposits (Gündoğdu et al. 2022; Hu et al. 2022; Xu et al. 2022a), but few have directly compared the effects caused by different sizes of plastic films, such as micro- versus meso-plastics. This is especially important if biodegradable plastics are to be considered as substitutes for conventional plastics (Qin et al. 2021), because it remains uncertain whether size has an effect on plastic toxicity.

Many unknowns in the underlying mechanisms of microplastic effects exist; to date, polymer additives have received little consideration, especially regarding biodegradable plastics. Chemical compositions of biodegradable plastics are often kept confidential by manufacturers, but evidence suggests that during degradation, these plastics can release toxic additives that may harm soil biota (Kim et al. 2020; Wang et al. 2022). Both conventional and biodegradable plastics often contain additives in the form of plasticisers, antioxidants, stabilisers and pigments that are integrated into the polymeric matrix during the manufacturing process to improve their functionality (Bejgarn et al. 2015; Hahladakis et al. 2018; Tang et al. 2023). Additives play a crucial role, especially in the case of polymers sourced from natural materials or microorganisms, as PHA and PLA are. This is due to these polymers having inherent limitations in terms of their physical properties, such as their ability to withstand high temperatures (Beach et al. 2013; Khan et al. 2017; Zimmermann et al. 2020; Cao et al. 2023). These additives are known to be leached from plastics, having negative impacts on soil ecosystems (Wang et al. 2013); phthalates, a common plasticising agent, have been observed to inhibit microbiological activity and be taken up by plants, upon being leached from PE and PP during natural weathering (Sun et al. 2015; Wang et al. 2016; Blasing and Amelung 2018). This highlights the need to understand the effect of leachate from biodegradable plastics, compared to that from conventional plastics, on soil ecosystems. Almost all microplastics in agricultural soils are considered “aged” (Gao et al. 2021); this has been evidenced in research (Li et al. 2020b; Yang et al. 2023) due to the noted visible cracks seen in field-collected plastics. Despite this, many previous studies have focused on the impact of “unaged”, also commonly known as “pristine” microplastics, on soil ecosystems (Qiu et al. 2022). Research on toxic effects of aged plastics, as opposed to pristine plastics, within soil ecosystems is less common, posing questions on whether it is the physical effect of the plastic or the chemical effect of the leachate additives having the discussed ecotoxicological impacts.

This study, therefore, was designed to assess the impacts of plastic contamination on the development of *Lolium perenne* (perennial ryegrass) and its soil environment. *L. perenne*, is known to be one of the most ecologically and agronomically important grass species in terms of pasture and forage in temperate regions, such as the UK, due to its high feed value and perenniality (Matzrafi et al. 2021) and is useful as a model species in ecotoxicology (Holmes 1980). The effects of micro- and meso-plastics, manufactured of polyethylene, polypropylene (both conventional types of plastic), polyhydroxybutyrate and polylactic acid (both biodegradable types of plastic) were assessed using mesocosm systems, providing controlled conditions. Three experiments tested the hypotheses that the addition of conventional and biodegradable micro- and meso-plastics, in either (i) pristine or (ii) aged or (iii) as leachate would alter the (a) shoot and root biomass, (b) chlorophyll-*a* and -*b* contents of *L. perenne* and (c) pH, organic matter content and respiration rate of the soil. The pristine experiment was designed to test both physical and chemical elements of plastic pollution, as pristine plastics leach out their chemical additives when naturally aged in the soil. In contrast, the aged and leachate experiments distinctively tested the physical and chemical elements of plastic pollution. The aged experiment specifically examined the physical presence of aged plastics, which inherently have fewer additives available to leach out due to their age. The leachate experiment focused on testing the effects of additive leaching.

## Materials and methods

### Experimental design and setup

Three separate mesocosm experiments were carried out using perennial ryegrass (*Lolium perenne*, Cotswold Grass Seeds Direct, UK), grown in topsoil (Westland Horticulture Ltd. UK). Experiment 1 used pristine micro- and meso-plastic films, Experiment 2 used aged micro- and meso-plastic films and Experiment 3 used leachate from micro- and meso-plastic without films present. Commercially available plastic films of PE, PP, PHB (0.01 mm thickness) and PLA (0.05 mm thickness) (Goodfellows, Cambridge, UK) were cut in micro (∼15 mm^2^) and meso (∼213 mm^2^) sizes (∼3.8 and ∼14.6 mm in side length, respectively) (Figure S1)—these approximate sizes were chosen as they are the median values of microplastic and meso-plastic size ranges (4–25 mm^2^ and 25–400 mm^2^, respectively) (Hartmann et al. 2019). The topsoil used was a rich clay loam soil with a pH of 6.05 ± 0.03 (mean ± SEM, *n* = 5) and an organic matter content of 20.6 ± 0.3% (mean ± SEM, *n* = 5). It is important to note that topsoil likely contains microplastics, given that they are sourced from the different locations within the environment and combined. The use of control samples with the same topsoil (with thorough homogenisation between bags) allows the comparison of treatment effects while acknowledging the possible presence of other microplastics in the substrate. For each separate mesocosm experiment—pristine, aged and leachate—there were two treatment factors “Polymer” and “Size”. Polymer had five levels: PE, PP, PHB, PLA and a shared Control, which had no added polymers. Size had two levels: micro and meso (see Figure S2 for more detail). All treatments were replicated five times (*n* = 5, *N* = 45).

For all experiments, the soil was air-dried, sieved (2-mm mesh size) and homogenised by hand. For Experiment 1, pristine plastics were added to soil at a concentration of 0.1% (w/w). Thus, 0.5 g of each plastic type was mixed into 500 g of soil for each mesocosm (polypropylene plant pot: 1.3 L capacity; height = 13.0 cm, top diameter = 12.5 cm, bottom diameter = 10.2 cm) to reach a dry bulk density of 1.1 g cm^−3^. Concentrations used to leach additives from plastics, simultaneously producing “aged” plastics, has varied remarkably among published studies (e.g. from 2 g L^−1^ (Lee et al. 2022) to 100 g L^−1^ (Esterhuizen et al. 2022); these values represent the mass of plastic per litre of water), but has been primarily based on measured concentrations of environmental plastic pollution in the field (Coffin et al. 2018; Bridson et al. 2021). The environmental aging of MP cannot be fully simulated by mechanical stress alone, as photodegradation also plays a significant role (Liu et al. 2021). To mimic plastic weathering by aging the plastics in an accelerated laboratory setting, 2.5 g each plastic was shaken in 1 L deionised water (a concentration of 0.25% (w/v), corresponding to the lower values in literature) at 120 rpm at 50 °C under a UV light, for 2 weeks (Rummel et al. 2019; Kim et al. 2020; Esterhuizen et al. 2022). From each leaching chamber, 0.5 g plastic in 200 mL water was extracted, with the plastic added to soil of Experiment 2, at a concentration of 0.1% (w/w), and the resulting leachate (the by-product from the aging process) used for Experiment 3, at a concentration of 0.25% (w/v). The leachate was added to each pot across 10 watering events (0.4% v/w): day 0 and then every 3 days until harvest at day 30 (20 mL leachate per watering event). Each mesocosm received approximately 100 (0.18 g) *L. perenne* seeds, a planting density of 0.81 seeds cm^−2^. The mesocosms were randomly assorted and periodically rotated at random to ensure uniformity in growth. The plants were grown for 30 days from 25/10/2022 until 24/11/2022 indoors next to a north-west-facing window and, under natural light conditions, received a daily photosynthetically active radiation average of 11.5 µmol m^−2^ s^−1^. For all experiments, soils were watered with deionised water to obtain 60% water holding capacity (WHC) at every watering event. WHC was determined gravimetrically from separate, dedicated pots.

### Above and below ground biomass and chlorophyll content of L. perenne

At the end of the experiment, shoots were cut at soil level and wet biomass was determined, along with dry biomass, after being oven-dried at 105 °C for 24 h. Plant roots were removed during a 5-min manual search per pot; roots were sieved, washed and dried at 105 °C to measure biomass. Prior to biomass analysis, samples for the measurement of chlorophyll content were prepared by extracting a subsample (0.2 g) of shoots from each mesocosm in 95% ethanol solution for 18 h in darkness. Chlorophyll-*a* and -*b* contents were determined by measuring the extractant at an absorbance of 664 nm and 647 nm, respectively, using a spectrophotometer (Jenway 6300 Spectrophotometer) (Harmut 1987; Wang et al. 2020). The chlorophyll concentrations were calculated following equations by Jeffrey and Humphrey (1975) (chlorophyll-*a*: 11.93 × *λ*_664nm_ − 1.93 × *λ*_647nm_ and chlorophyll-*b*: 20.36 × *λ*_647nm_ − 5.50 × *λ*_664nm_).

### Soil pH, organic matter and respiration measurements

Soil pH was determined using a Hanna HI 991300 pH meter at a soil to water ratio of 1:1, after mechanical shaking for 1 h and centrifuging at 3000 g for 3 min (Rowell 1994). Soil organic matter content was determined by calculating the loss on ignition: 10 g soil was oven-dried at 105 °C for 18 h to achieve a constant dry weight and then was combusted at 350 °C for 12 h in a muffle furnace and reweighed. The weight loss during ignition in the furnace is proportional to the quantity of organic matter in the sample (Rowell 1994). Soil respiration rates were measured following plant harvest methods in Rowell (1994): moist soil was incubated with NaOH at 20 °C for 1 week. Following this, BaCl_2_ and deionised water were added to the NaOH and titrated against HCl, using phenolphthalein indicator.

### Statistical analysis

**Fig. 1:**
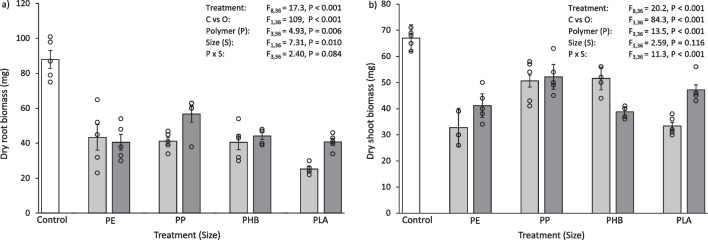
**a** Dry root biomass (mg) and **b** dry shoot biomass (mg) of *L. perenne* after 30 days exposure to 0.1% (w/w) pristine micro- and meso-plastics. The white bars (left) represent the control; light grey bars represent microplastic treatments; dark grey bars represent meso-plastic treatments. Data are means (± SEM, *n* = 5), the superimposed dots represent the raw data and ANOVA results are included (C vs O = Control vs Others)

**Fig. 2:**
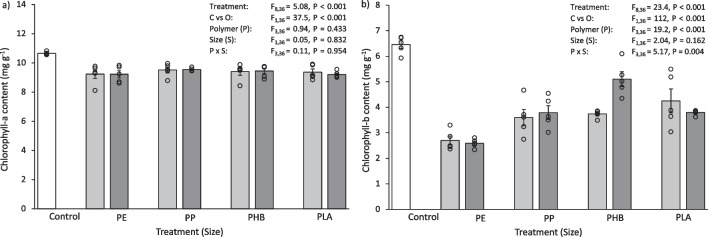
**a** Chlorophyll-*a* content (mg g^−1^ dry biomass) and **b** chlorophyll-*b* content (mg g^−1^ dry biomass) of *L. perenne* after 30 days exposure to 0.1% (w/w) pristine micro- and meso-plastics. The white bars (left) represent the control; light grey bars represent microplastic treatments; dark grey bars represent meso-plastic treatments. Data are means (± SEM, *n* = 5), the superimposed dots represent the raw data and ANOVA results are included (C vs O = Control vs Others)

**Fig. 3:**
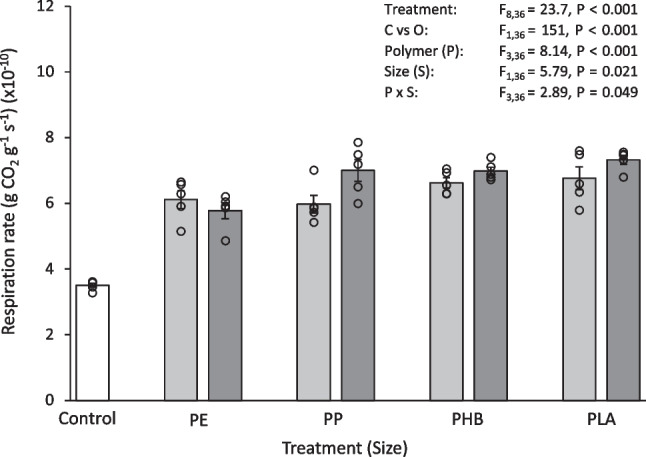
Respiration rate (gCO_2_ g^−1^ s^−1^) (× 10^−10^) of soil after 30 days exposure to 0.1% (w/w) pristine micro- and meso-plastics. The white bars (left) represent the control; light grey bars represent microplastic treatments; dark grey bars represent meso-plastic treatments. Data are means (± SEM, *n* = 5), the superimposed dots represent the raw data and ANOVA results are included (C vs O = Control vs Others)

**Fig. 4:**
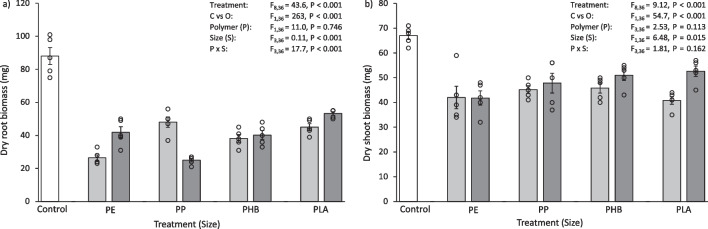
**a** Dry root biomass (mg) and **b** dry shoot biomass (mg) of *L. perenne* after 30 days exposure to 0.1% (w/w) aged micro- and meso-plastics. The white bars (left) represent the control; light grey bars represent microplastic treatments; dark grey bars represent meso-plastic treatments. Data are means (± SEM, *n* = 5), the superimposed dots represent the raw data and ANOVA results are included (C vs O = Control vs Others)

**Fig. 5:**
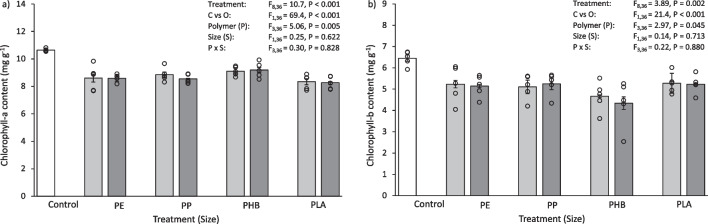
**a** Chlorophyll-*a* content (mg g^−1^ dry biomass) and **b** chlorophyll-*b* content (mg g^−1^ dry biomass) of *L. perenne* after 30 days exposure to 0.1% (w/w) aged micro- and meso-plastics. The white bars (left) represent the control; light grey bars represent microplastic treatments; dark grey bars represent meso-plastic treatments. Data are means (± SEM, *n* = 5), the superimposed dots represent the raw data and ANOVA results are included (C vs O = Control vs Others)

**Fig. 6:**
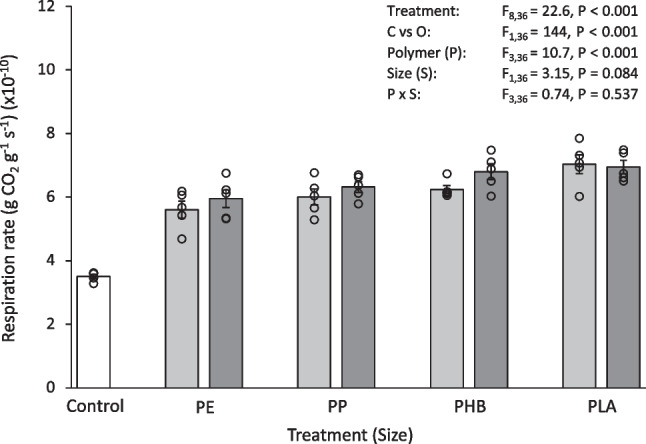
Respiration rate (gCO_2_ g^−1^ s^−1^) (× 10^−10^) of soil after 30 days exposure to 0.1% (w/w) aged micro- and meso-plastics. The white bars (left) represent the control; light grey bars represent microplastic treatments; dark grey bars represent meso-plastic treatments. Data are means (± SEM, *n* = 5), the superimposed dots represent the raw data and ANOVA results are included (C vs O = Control vs Others)

**Fig. 7:**
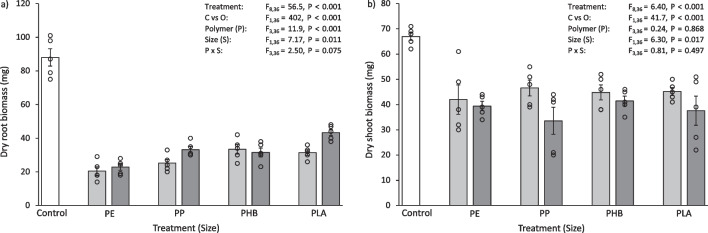
**a** Dry root biomass (mg) and **b** dry shoot biomass (mg) of *L. perenne* after 30 days exposure to plastic leachate. The white bars (left) represent the control; light grey bars represent microplastic treatments; dark grey bars represent meso-plastic treatments. Data are means (± SEM, *n* = 5), the superimposed dots represent the raw data and ANOVA results are included (C vs O = Control vs Others)

**Fig. 8:**
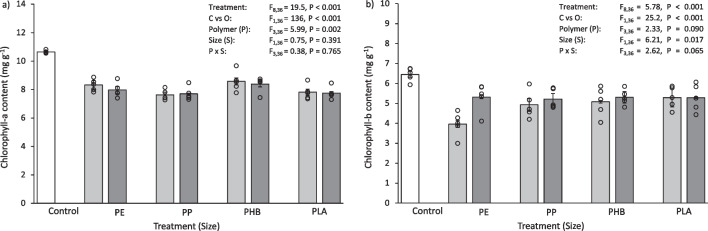
**a** Chlorophyll-*a* content (mg g^−1^ dry biomass) and **b** chlorophyll-*b* content (mg g^−1^ dry biomass) of *L. perenne* after 30 days exposure to micro- and meso-plastic leachates. The white bars (left) represent the control; light grey bars represent microplastic treatments; dark grey bars represent meso-plastic treatments. Data are means (± SEM, *n* = 5), the superimposed dots represent the raw data and ANOVA results are included (C vs O = Control vs Others)

**Fig. 9:**
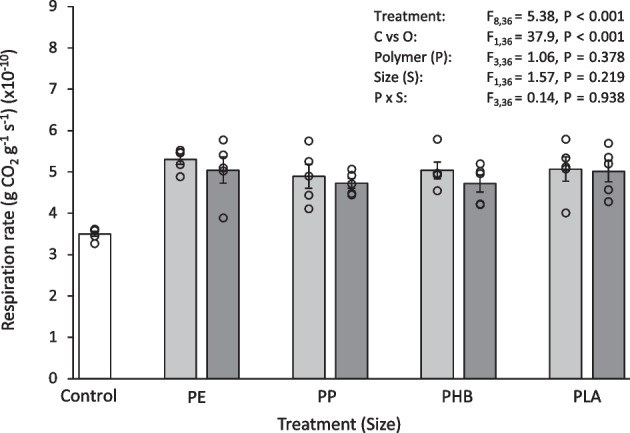
Respiration rate (gCO_2_ g^−1^ s^−1^) (× 10^−10^) of soil after 30 days exposure to plastic leachate. The white bars (left) represent the control; light grey bars represent microplastic treatments; dark grey bars represent meso-plastic treatments. Data are means (± SEM, *n* = 5), the superimposed dots represent the raw data and ANOVA results are included (C vs O = Control vs Others)

The data analysis followed Green et al. (2016). Using R v 4.1.0 (R Core Team 2021), normality, homoscedasticity and equality of variance were tested using Shapiro-Wilkinson tests, residual plots and Levene’s tests (from the car package (Fox and Weisberg 2018)), respectively. For each mesocosm experiment, the experimental design was asymmetric with two orthogonal factors “Polymer” and “Size”, with a single control group “Control”. Therefore, results for each experiment were analysed by using the mean squares from two independent analyses of variance (ANOVA), which involved the partitioning of variance for individual mesocosm experiments (pristine plastics, aged plastics and plastic leachates). Firstly, a one-way ANOVA with all treatments as separate levels (*a* = 9, *n* = 5, *N* = 45) was calculated, followed by a two-way ANOVA of “*Polymer*” by “*Size*” (*P* × *S*) with the level “Control” removed from the dataset (*a* = 4, *b* = 2, *n* = 5, *N* = 40). The 1st ANOVA produced residuals which were then used to estimate any differences between “*Polymer*” and “*Size*” in the 2nd ANOVA, enabling the determination of any variation between the Control and the other treatments (“Control versus Others”), contrasted at one degree of freedom (Underwood 1997). When a significant effect (at *P* < 0.05) was found in the “Control versus Others” contrast, a Dunnett’s test (from the DescTools package (Signorell et al. 2021)) was used to determine where the significant difference existed by contrasting the Control with each level of the significant term. When the main terms were significant (at *P* < 0.05), Tukey HSD tests were used for pairwise comparisons between the “*Polymer*” and “*Size*” in the 2nd ANOVA. The in-text results are described as a percentage change, as a measure of effect size, and are presented in Table 1, 2, and 3 and Figs. 1, 2, 3, 4, 5, 6, 7, 8, and 9.

**Table 1:** Soil physicochemical characteristics pH and loss on ignition (LOI) as an estimation for soil organic matter content after 30 days exposure to 0.1% (w/w) pristine micro- and meso-plastics. Data are means (± SEM, *n* = 5) and ANOVA results are included

Treatment	pH	LOI (%)
Control	6.15 ± 0.03	18.1 ± 0.1
Micro PE	4.81 ± 0.04	9.0 ± 0.3
Meso PE	5.00 ± 0.03	7.6 ± 1.9
Micro PP	4.85 ± 0.04	11.7 ± 0.5
Meso PP	5.08 ± 0.01	14.5 ± 0.9
Micro PHB	5.16 ± 0.05	14.1 ± 0.2
Meso PHB	5.47 ± 0.05	14.4 ± 0.7
Micro PLA	5.35 ± 0.04	15.1 ± 0.4
Meso PLA	5.41 ± 0.05	14.6 ± 0.4
Source of variation		
Treatment	F_8,36_ = 98.0, *P* < 0.001	F_8,36_ = 57.8, *P* < 0.001
Control vs Others	F_1,36_ = 527, *P* < 0.001	F_1,36_ = 159, *P* < 0.001
Polymer (*P*)	F_1,36_ = 67.7, *P* < 0.001	F_1,36_ = 87.8, *P* < 0.001
Size (*S*)	F_3,36_ = 45.6, *P* < 0.001	F_3,36_ = 11.2, *P* = 0.002
*P* vs *S*	F_3,36_ = 3.03, *P* = 0.042	F_3,36_ = 9.45, *P* < 0.001

**Table 2:** Soil physicochemical characteristics pH and loss on ignition (LOI) as an estimation for soil organic matter content after 30 days exposure to 0.1% (w/w) aged micro- and meso-plastics. Data are means (± SEM, *n* = 5) and ANOVA results are included

Treatment	pH	LOI (%)
Control	6.15 ± 0.03	18.1 ± 0.1
Micro PE	5.58 ± 0.03	13.2 ± 0.3
Meso PE	5.67 ± 0.03	13.6 ± 0.6
Micro PP	5.68 ± 0.04	14.1 ± 0.3
Meso PP	5.74 ± 0.02	13.8 ± 0.4
Micro PHB	5.84 ± 0.04	17.9 ± 2.3
Meso PHB	5.59 ± 0.02	14.9 ± 0.2
Micro PLA	5.42 ± 0.05	14.6 ± 0.4
Meso PLA	5.45 ± 0.05	14.1 ± 0.8
Source of variation		
Treatment	F_8,36_ = 40.4, *P* < 0.001	F_8,36_ = 11.8, *P* < 0.001
Control vs Others	F_1,36_ = 207, *P* < 0.001	F_1,36_ = 81.9, *P* < 0.001
Polymer (*P*)	F_3,36_ = 28.3, *P* < 0.001	F_3,36_ = 1.68, *P* = 0.188
Size (*S*)	F_1,36_ = 0.64, *P* = 0.431	F_1,36_ = 1.03, *P* = 0.318
*P* vs *S*	F_3,36_ = 10.3, *P* < 0.001	F_3,36_ = 2.17, *P* = 0.109

**Table 3:** Soil physicochemical characteristics pH and loss on ignition (LOI) as an estimation for soil organic matter content after 30 days exposure to 0.25% (w/v) micro- and meso-plastic leachate. Data are means (± SEM, *n* = 5) and ANOVA results are included

Treatment	pH	LOI (%)
Control	6.15 ± 0.03	18.1 ± 0.1
Micro PE	5.62 ± 0.06	11.7 ± 0.4
Meso PE	5.66 ± 0.05	11.4 ± 0.2
Micro PP	5.63 ± 0.06	12.2 ± 0.4
Meso PP	5.67 ± 0.05	12.0 ± 0.3
Micro PHB	5.71 ± 0.04	14.4 ± 0.3
Meso PHB	5.93 ± 0.03	14.2 ± 0.2
Micro PLA	5.70 ± 0.04	13.0 ± 0.2
Meso PLA	5.69 ± 0.05	12.5 ± 0.4
Source of variation		
Treatment	F_8,36_ = 14.2, *P* < 0.001	F_8,36_ = 45.2, *P* < 0.001
Control vs Others	F_1,36_ = 82.9, *P* < 0.001	F_1,36_ = 271, *P* < 0.001
Polymer (*P*)	F_3,36_ = 6.08, *P* = 0.002	F_3,36_ = 29.8, *P* < 0.001
Size (*S*)	F_1,36_ = 4.97, *P* = 0.032	F_1,36_ = 1.66, *P* = 0.205
*P* vs *S*	F_3,36_ = 2.43, *P* = 0.081	F_3,36_ = 0.09, *P* = 0.963

## Results

### Effects of pristine plastics

#### Biomass and chlorophyll content of L. perenne

Overall, the plants grown in the control had a greater biomass compared to those exposed to pristine plastics. The dry biomass of *L. perenne* roots after 30 days exposure to pristine plastics was 35–71% less than the control (Fig. 1a), which was significantly different for all treatments (Control vs Others, F_1,36_ = 109, *P* < 0.001). Mirroring this, the dry biomass of *L. perenne* shoots was 22–51% less than the control (Fig. 1b). This was also significantly less than the control for all treatments (Control vs Others, F_1,36_ = 109, *P* < 0.001). The type of plastic added to the soil influenced shoot biomass. In particular, the shoot biomass of *L. perenne* when exposed to microPE was significantly lower than some other treatments (microPE vs microPP, microPHB, *P* < 0.001), with the microPE treated soil having a 35–36% less shoot biomass than the compared treatments. Some other significant differences in shoot biomass were found between *Size*, micro and meso, and *Polymer*, PE, PP, PHB and PLA (Table S1).

Plants grown in the control group had more chlorophyll than those exposed to pristine plastics. The chlorophyll-*a* content of *L. perenne* shoots after exposure to pristine plastics was 10–14% less than the control (Fig. 2a), which was significantly different for all treatments (Control vs Others, F_1,36_ = 112, *P* < 0.001). Similarly, the chlorophyll-*b* content of *L. perenne* shoots after 30 days exposure to pristine plastics was 21–60% less than the control (Fig. 2b). This was also significantly less than the control for all treatments (Control vs Others, F_1,36_ = 112, *P* < 0.001). The type of plastic added to the soil influenced chlorophyll-*b* content. In particular, the chlorophyll-*b* content of *L. perenne* when exposed to microPE and mesoPE was significantly lower than some other treatments (microPE vs microPLA, *P* = 0.003; mesoPE vs mesoPP, mesoPHA, mesoPLA, *P* =  < 0.001–0.041), with the microPE treated soil having a 37% lower chlorophyll-*b* content and the mesoPE treated soil having a 32–49% lower chlorophyll-*b* content than the compared treatments. Some other significant differences in chlorophyll-*b* content were found between *Size*, micro and meso, and *Polymer*, PE, PP, PHB and PLA (Table S1).

#### Soil pH, organic matter and respiration rate

Overall, the control soil had a greater pH and organic matter content and a lower respiration rate than the soils exposed to pristine plastics. Soil pH after 30 days exposure to pristine plastics was 0.68–1.34 units (12–22%) lower than the control (Table 1), which was significantly different for all treatments (Control vs Others, F_1,36_ = 527, *P* < 0.001). After 30 days exposure to pristine plastics, soil organic matter content was 16–58% lower than the control (Table 1). This was also significantly less than the control for all treatments (Control vs Others, F_1,36_ = 159, *P* < 0.001). The type of plastic added to the soil also influenced organic matter content. In particular, the organic matter content of soil exposed to microPE and microPP was significantly less than some of the other treatments (microPE vs microPP, microPHB, microPLA, *P* < 0.001; microPP vs microPHB, microPLA, *P* =  < 0.001–0.015), with the microPE treated soil having 23–40% less soil organic matter and microPP treatment having 17–23% less soil organic matter than the compared treatments. The respiration rate of soil after exposure to pristine plastics was 39–52% higher than the control (Fig. 3) which was significantly different for all treatments (Control vs Others, F_1,36_ = 151, *P* < 0.001). Some significant differences in soil pH, organic matter content and respiration rate were found between *Size*, micro and meso, and *Polymer*, PE, PP, PHB and PLA (Table S1).

### Effects of aged plastics

#### Biomass and chlorophyll content of L. perenne

Overall, the plants grown in the control had a greater biomass compared to those exposed to aged plastics. The dry biomass of *L. perenne* roots after 30 days exposure to aged plastics was 45–71% less than the control (Fig. 4a), which was significantly different for all treatments (Control vs Others, F_1,36_ = 263, *P* < 0.001). Correspondingly, the dry biomass of *L. perenne* shoots after 30 days exposure to aged plastics was 21–39% lower than the control (Fig. 4b). This was also significantly less than the control for all treatments (Control vs Others, F_1,36_ = 54.7, *P* < 0.001). The type of plastic added to the soil influenced root biomass. In particular, the root biomass of *L. perenne* when exposed to microPE was significantly lower than some other treatments (microPE vs microPP, microPHB, microPLA, *P* < 0.001–0.025), with the microPE treated soil having 31–45% less root biomass. Plants exposed to mesoPE and mesoPP also had a significantly lower root biomass than some other treatments (mesoPE vs mesoPLA, *P* < 0.026; mesoPP vs mesoPHB, mesoPLA, *P* < 0.001–0.002), with the PE treated soil having 21% less biomass and the PP treated soil having 38–53% less root biomass than the compared treatments. Some other significant differences in root biomass were found between *Size*, micro and meso, and *Polymer*, PE, PP, PHB and PLA (Table S2).

The plants grown in the control group had more chlorophyll than those exposed to aged plastics. The chlorophyll-*a* content of *L. perenne* shoots after 30 days exposure to aged plastics was 17–22% lower than the control (Fig. 5a), which was significantly different for all treatments (Control vs Others F_1,36_ = 69.4, *P* < 0.001). The chlorophyll-*b* content of *L. perenne* shoots after 30 days exposure to aged plastics was 18–33% lower than the control (Fig. 5b). This was also significantly less than the control for all treatments (Control vs Others, F_1,36_ = 21.4, *P* < 0.001).

#### Soil pH, organic matter and respiration rate

In summary, the control soil had a greater pH and organic matter content and a lower respiration rate than the soils exposed to aged plastics. Soil pH after 30 days exposure to aged plastics was 0.31–0.73 units (5–12%) lower than the control (Table 2), which was significantly different for all treatments (Control vs Others, F_1,36_ = 207, *P* < 0.001). Soil organic matter content after exposure to aged plastics was 1–27% less than the control (Table 2). This was also significantly less than the control for all treatments (Control vs Others, F_1,36_ = 81.9, *P* < 0.001). The respiration rate of soil after 30 days exposure to aged plastics was 37–50% higher than the control (Fig. 6) which was significantly different for all treatments (Control vs Others, F_1,36_ = 144, *P* < 0.001). Some significant differences in soil pH, organic matter content and respiration rate were found between *Polymer*, PE, PP, PHB and PLA (Table S2).

### Effects of leachate from plastics

#### Biomass and chlorophyll content of L. perenne

The plants grown in the control had a greater biomass compared to those exposed to plastic leachate. The dry biomass of *L. perenne* roots after 30 days exposure to plastic leachate was 51–77% less than the control (Fig. 7a), which was significantly different for all treatments (Control vs Others, F_1,36_ = 402, *P* < 0.001). Similarly, the dry biomass of *L. perenne* shoots after 30 days exposure to plastic leachate was 30–50% less than the control (Fig. 7b). This was also significantly less than the control for all treatments (Control vs Others, F_1,36_ = 41.7, *P* < 0.001). The type of plastic added to the soil influenced root biomass. In particular, the root biomass of *L. perenne* when exposed to microPE and mesoPE was significantly lower than some other treatments (microPE vs microPHB, microPLA, *P* = 0.006–0.030; mesoPE vs mesoPP, mesoPLA, *P* < 0.001–0.047), with the microPE treated soil having 35–39% less root biomass and the mesoPE treated soil having 31–47% less root biomass than the compared treatments. Some other significant differences in root biomass were found between *Size*, micro and meso, and *Polymer*, PE, PP, PHB and PLA (Table S3).

Plants grown in the control had more chlorophyll compared to those exposed to plastic leachate. The chlorophyll-*a* content of *L. perenne* shoots after 30 days exposure to plastic leachate was 19–28% lower than the control (Fig. 8a), which was significantly different for all treatments (Control vs Others, F_1,36_ = 136, *P* < 0.001). Similarly, the chlorophyll-*b* content of *L. perenne* shoots after 30 days exposure to plastic leachate was 18–39% lower than the control (Fig. 8b). This was also significantly less than the control for all treatments (Control vs Others, F_1,36_ = 25.2, *P* < 0.001). The type of plastic added to the soil influenced chlorophyll-*b* content. In particular, the chlorophyll-*b* content of *L. perenne* when exposed to microPE was significantly different to microPLA (microPE vs microPLA, *P* < 0.001), with the microPE treated soil having a 25% lower chlorophyll-*b* content than microPLA. Some other significant differences in chlorophyll-*b* content were found between *Size*, micro and meso, and *Polymer*, PE, PP, PHB and PLA (Table S3).

#### Soil pH, organic matter and respiration rate

Overall, the control soil had a greater pH and organic matter content and a lower respiration rate than the soils exposed to plastic leachates. Soil pH after 30 days exposure to plastic leachate was 0.22–0.53 units (4–9%) lower than the control (Table 3), which was significantly different for all treatments (Control vs Others, F_1,36_ = 82.9, *P* < 0.001). Soil organic matter content after 30 days exposure to plastic leachate was 21–37% less than the control (Table 3). This was also significantly less than the control for all treatments (Control vs Others, F_1,36_ = 271, *P* < 0.001). The type of plastic added to the soil also influenced organic matter content. In particular, the organic matter content of soil exposed to microPHB was significantly different to some other treatments (microPHB vs microPE, microPP, *P* < 0.001), with the PHB-treated soil having an 18–23% higher organic matter content than the compared treatments. The respiration rate of soil after 30 days exposure to plastic leachate was 26–34% higher than the control (Fig. 9) which was significantly different for all treatments (Control vs Others, F_1,36_ = 37.9, *P* < 0.001). Some significant differences in soil pH and organic matter content were found between *Size*, micro and meso, and *Polymer*, PE, PP, PHB and PLA (Table S3).

## Discussion

The effects of plastic contamination on shoot and root biomass and chlorophyll content of *L. perenne* and soil pH, organic matter content and respiration rate were indistinguishable based on the state of the plastics (pristine, aged or leachate), indicating that both the physical plastic and the chemical leachate play a role in the observed effects.

### Responses of L. perenne to different types, sizes and age of plastics in the soil

Several growth responses of *L. perenne* were altered when plastics manufactured of PE, PP, PHB and PLA in physical and chemical forms were incorporated into the soil matrix. A decrease in root biomass could compromise the ability of plants to obtain water and necessary nutrients from the soil. Recent studies have focused on the ability of plants to internalise plastic particles. Li et al. (2020a) demonstrated that *Triticum aestivum* (wheat) and *Lactuca sativa* (lettuce) can uptake nanobeads (200 nm) and microbeads (2.0 µm) from the root to the shoot through transpirational pull. This may be possible if the plastics in the present study have begun to degrade into smaller pieces in the soil. Due to the size of the plastics used in the present study, however (microplastics, ~ 3.8 mm, and meso-plastics, ∼14.6 mm in length), root entanglement with films is more probable, and this may impede root development. Plastic mulch films have been recognised for their potential to become entangled with plant roots, posing challenges in post-harvest removal (Zhao et al. 2017; Li et al. 2022). This entanglement not only presents difficulties during removal but also holds the potential to impede root development, as evidenced by the decreased root biomass of *L. perenne* grown in soils exposed to pristine and aged plastics. In addition to the complications arising from root entanglement, our study reveals an additional impact on plant development. Specifically, when examining *L. perenne* exposed to plastic leachate, a distinct reduction in root biomass was observed. This outcome suggests that the influence of plastic on plant performance extends beyond the physical entanglement of roots; the presence of plastic additives is observed to be detrimental to soil ecosystems, underscoring a multifaceted influence of plastic exposure on plant growth.

The current body of literature places a limited emphasis on the impacts of biodegradable leachates on plants, with a more predominant focus on conventional plastics, particularly in terms of phthalate esters, bisphenol A, nonylphenol compounds and polybrominated diphenyl ethers (Cao et al. 2023). The presence of phthalate esters in soil, for example, has been shown to reduce the contents of total phosphorus, total nitrogen, and available potassium (Cao et al. 2023), inducing phytotoxic effects, decreasing germination rates and inhibiting root development of plants (Zou et al. 2017; Gao et al. 2021). In a study by Esterhuizen et al. (2022), *Lolium multiflorum* (Italian ryegrass) planted in soil containing (3% w/w) pristine and naturally aged (high-density) PE fragments (4 mm), and their leachates (0.44% w/v), resulted in roots and shoots with a 77–83% lower fresh weight than the controls. Similar to the results of the present study, Esterhuizen et al. (2022) found the inhibition of root and shoot growth to be comparable, irrespective of exposure to particles or leachates of the same plastic. Esterhuizen et al. (2022), however, also looked at artificially aged (high-density) PE fragments, finding the difference between new and aged microplastics and leachates to indicate that the aging of the plastic significantly decreases the toxicological effect on root and shoot growth. These results, as well as those conducted by others within their group (Pflugmacher et al. 2020, 2021a, 2021b), indicate a correlation between aging and a decrease in phytotoxicity, conflicting the results of the present study, where the suppression of root and shoot biomass was similar, regardless of whether the exposure was to pristine or aged films of the same polymer. *L. perenne* exposed to conventional PE films in pristine, aged and leachate forms had significantly lower biomasses than some of the biodegradable PHB and PLA treatments.

Changes in the contents of photosynthetic pigments, such as chlorophylls, are commonly used as biomarkers to indicate plant stress (Pavlović et al. 2014). Yang and Gao (2022) found the effects of conventional and biodegradable microplastics from mulch films to both inhibit photosynthetic rate and chlorophyll content of *Oryza sativa* (rice). Notably, PE exhibited a greater negative effect than polybutylene adipate terephthalate (PBAT). Similarly, in the present study, despite all plastic forms and types repressing chlorophyll content, *L. perenne* exposed to conventional PE films in pristine and leachate forms had significantly lower chlorophyll-*b* contents than some of the biodegradable PHB and PLA treatments. Xu et al. (2022b) found conventional microplastics to cause a significant decrease in chlorophyll-*a* and -*b* content by accelerating the breakdown of chlorophyll through its conversion to phytol. Chlorophyll-*a* serves as the primary pigment in the reaction centres, while chlorophyll-*b* acts as an accessory pigment, broadening the range of light that can be utilised for the synthesis of organic compounds, subsequently enhancing the efficiency of photosynthesis (Katz et al. 1978; Björn et al. 2009). Chlorophyll is therefore essential in the primary production of agroecosystems and maintaining a stable state of chlorophylls is essential for the process of photosynthesis in plants (Wang et al. 2020). When exposed to plastic particles and plastic leachates, plants undergo a state of heightened stress, having potentially detrimental causal effects at the ecosystem level.

In this experiment, the thickness of the PLA (0.05 mm) used was greater than that of the PE, PP and PHA (0.01 mm). This meant that the surface area of the PLA was 2% greater than the other polymers for microplastics and 0.5% greater for meso-plastics. Soils treated with PE, PP and PHA therefore had approximately 5–7 times more plastic pieces within the 0.5 g addition than those treated with PLA, based on the density of the plastics and the known masses used (Table S4). This difference may elucidate the significant differences in respective chlorophyll-*b* content and biomass of *L. perenne* grown in soil treated with pristine and aged PLA, in contrast to conventional plastics. Notably, these differences were also observed between conventional plastics and PHA. Despite PHB and the conventional plastics sharing the same thickness, PHB exhibits a greater density than these plastics, with 1.3–1.4 times more plastic pieces within the 0.5 g addition compared to those treated with PE and PP. The exploration of the effects of plastic film thickness and density on soil ecosystems is currently understudied, and the findings of the current study present an opportunity for further research to investigate plastic film thickness and density as an independent variable.

### Effects of different types, sizes and age of plastics on soil physico-chemical properties

A lowered soil pH has also been reported by Wang et al. (2020), who found a reduction in pH with increasing HDPE from 0.1 to 1.0%. However, Wang et al. (2020) found soil pH to increase with increasing PLA dose (0.1 to 10%), whereas Boots et al. (2019) found the incorporation of PLA (0.1% w/w) to have no influence on soil pH, contrasting the results of the present study, which found biodegradable plastics to also lower soil pH. Mortula et al. (2021) found that a low pH is destructive to plastics, promoting leaching, which, in turn, could create a feedback loop: a lowered soil pH due to the presence of plastics causes more plastic leaching which further lowers the soil pH, as the present study found both particles and leachates to decrease soil pH. The availability of nutrients to plants through the solubility of nutrients in the soil solution is impacted by soil pH. A decrease in soil pH can also cause the immobilisation of plant nutrients, leading to a delayed nutrient release to the plant (Souza and Billings 2022). The decrease in soil pH may therefore cause plant stress, which is demonstrated by the reduction in *L. perenne* biomass and chlorophyll content, as seen in the present study.

This study found all polymer forms and types to reduce soil organic matter, but soil exposed to conventional films in pristine and leachate forms had a lower organic matter content compared to some of the biodegradable treated soils. In the pristine and leachate experiments, some conventional plastics caused a greater decrease in soil organic matter content than biodegradable plastic treatments. Liu et al. (2023b) report in their meta-analysis (168 publication observations) that the presence of non-biodegradable microplastics increases soil respiration by 18%, which suggests that these conventional plastics have the potential to give rise to the soil organic carbon loss. This is confirmed in the present study by the decrease in organic matter content and increase in respiration rate in soils treated with both non-biodegradable (conventional) and biodegradable plastics and their leachates. Biodegradation of plastics is usually assessed by measuring the conversion of organic carbon into CO_2_ (Sander 2019), an observation potentially elucidated by the findings presented here. Due to the conventional and biodegradable plastics exhibiting comparable effects in terms of these measured soil physico-chemical parameters, it could be inferred that the degradation of bioplastics has not initiated. This might be attributed to regulation by a limiting environmental factor, such as temperature, moisture levels and the presence of plastic-degrading bacteria (Brodhagen et al. 2015). A possible pathway for the increased CO_2_ flux from the soil, indicated by the increased respiration rate, could be altered microbial activity. The decrease in soil pH observed in plastic-treated soils, relative to the control, may alter microbial biomass and activity (Pietri and Brookes 2008), in this case, potentially enhancing these microbial properties, which could contribute to the observed decrease in soil organic matter content. Soil microbes play a critical role in ecological processes such as the biogeochemical cycling of vital nutrients crucial for plant growth, as well as the decomposition of soil organic matter, potentially also influencing the decreased growth parameters seen in the present study (Yan et al. 2017a, b; Kang et al. 2021). Rillig et al. (2019) hypothesise that plastics affect plant growth by changing soil properties, impacting water availability and microbial activity. The results from the present study suggest that this mechanism is a likely cause of the impact on soil ecosystems, particularly on plant growth and stress. Soil organic matter is typically protected within soil aggregates, which physically shield the organic matter from degradation. The presence of microplastics may indirectly compromise the protective ability of soil aggregates by influencing soil structure and microbial activity, as suggested by de Souza Machado et al. (2018) and Boots et al. (2019).

### Wider implications and recommendations

This research contributes to the existing body of evidence, highlighting several potentially detrimental physical and chemical effects of plastics in terrestrial ecosystems, using a model system based on *L. perenne*. Conventional and biodegradable plastics have both physical and chemical impacts on essential soil characteristics and the growth of *L. perenne*, potentially leading to wider effects on soil ecosystem functioning and adding to the growing body of evidence highlighting the negative consequences of biodegradable plastic pollution in terrestrial environments. In general, meso (> 5 mm) and micro (< 5 mm) plastic films did not differ in the impact on plants or soil. The key finding of this work is that the effects of conventional and biodegradable plastics on plant and soil properties were indistinguishable based on the state of the plastics (pristine, aged or leachate), indicating that both the physical plastic and the chemical leachate play a role in the observed effects. This highlights the importance of accounting for plastic leachate when evaluating the potential impacts of plastic pollution on soil and plant health, emphasising the need for further research to examine the toxicological impacts of specific conventional, and more importantly, biodegradable plastic additives. These effects may have significant implications for crop quality and production in agriculture, as it is now known that physical plastics and their leachates can impact plant development and alter the surrounding soil environment. As the application of plastic mulching increases, this study underscores the need for a more thorough understanding of the potential risks associated with biodegradable plastics (Qin et al. 2021), as observed with conventional plastics (Qiang et al. 2023), particularly with regard to their leaching properties, before they are more widely adopted in agriculture.

### Supplementary Information

Below is the link to the electronic supplementary material.

Supplementary file1 (DOCX 22806 KB)

## Data Availability

Data is available on request.
