# Supplementary material for: Impacts of pristine, aged and leachate of conventional and biodegradable plastics on plant growth and soil organic carbon
Source: Environ Sci Pollut Res Int. 2024 Jan 15;31(8):11766–80. doi: 10.1007/s11356-024-31838-9 (PMC10869392; doi:10.1007/s11356-024-31838-9)
Supplement: Supplementary file 1 — Supplementary file1 (DOCX 22806 KB) [file 11356_2024_31838_MOESM1_ESM.docx]

**Supplementary Information**

Figure S1. Example of plastics, showing micro (top) and meso (bottom) sizes of PE, PP, PHB and PLA polymers (left to right), with a centimetre scale.

Figure S2. Diagrammatic representation of the experimental design, highlighting asymmetry due to a single control for both factors (POLYMER and SIZE) with equal replication, N = 45.

Table S1. Results of the two-way ANOVA for each response variable of the pristine plastic experiment. The data are organised following the structure of the manuscript, including a reference to corresponding tables or figures in the main manuscript. Different lower-case letters represent a significant difference between polymer treatments (Micro: a – c; Meso: p – r); different upper-case letters represent a significant difference between different sizes of the same polymer (p ≤ 0.05, ANOVA and Tukey test).

| Polymer type | Micro | Meso |
| --- | --- | --- |
| *3.2.1 Biomass and chlorophyll content of* L. perenne | | |
| Variable: Shoot biomass of *L. perenne* (Figure 1) | | |
| PE | 32.8±2.6^aA^ | 41.2±2.7^pqA^ |
| PP | 50.6±3.6^bA^ | 52.2±3.1^pB^ |
| PHB | 51.6±2.3^bA^ | 38.8±1.0^qB^ |
| PLA | 33.4±1.5^aA^ | 47.2±2.3^pqB^ |
| Variable: Chlorophyll-b content of *L. perenne* (Figure 2) | | |
| PE | 2.70±0.17^aA^ | 2.59±0.08^pA^ |
| PP | 3.60±0.32^abA^ | 3.79±0.27^qA^ |
| PHB | 3.74±0.07^abA^ | 5.11±0.29^rB^ |
| PLA | 4.25±0.47^aA^ | 3.79±0.05^qA^ |
| *3.2.2 Soil pH, organic matter and respiration rate* | | |
| Variable: Soil pH (Table 1) | | |
| PE | 4.81±0.04^aA^ | 5.00±0.03^pA^ |
| PP | 4.85±0.04^aA^ | 5.08±0.01^pB^ |
| PHB | 5.16±0.05^bA^ | 5.47±0.05^qB^ |
| PLA | 5.35±0.04^bA^ | 5.41±0.05^qA^ |
| Variable: Soil organic matter (Table 1) | | |
| PE | 9.0 ± 0.3^aA^ | 7.6 ± 1.9^pA^ |
| PP | 11.7 ± 0.5^bA^ | 14.5 ± 0.9^qB^ |
| PHB | 14.1 ± 0.2^cA^ | 14.4 ± 0.7^qA^ |
| PLA | 15.1 ± 0.4^cA^ | 14.6 ± 0.4^qA^ |
| Variable: Soil respiration rate (Figure 3) | | |
| PE | 6.11 ± 0.27^aA^ | 5.77 ± 0.24^pA^ |
| PP | 5.98 ± 0.27^aA^ | 7.00 ± 0.34^qA^ |
| PHB | 6.62 ± 0.16^aA^ | 6.98 ± 0.12^qA^ |
| PLA | 6.76 ± 0.34^aA^ | 7.32 ± 0.14^qA^ |

Table S2. Results of the two-way ANOVA for each response variable of the aged plastic experiment. The data are organised following the structure of the manuscript, including a reference to corresponding tables or figures in the main manuscript. Different lower-case letters represent a significant difference between polymer treatments (Micro: a – c; Meso: p – r); different upper-case letters represent a significant difference between different sizes of the same polymer (p ≤ 0.05, ANOVA and Tukey test).

| Polymer type | Micro | Meso |
| --- | --- | --- |
| *3.3.1 Biomass and chlorophyll content of* L. perenne | | |
| Variable: Root biomass of *L. perenne* (Figure 4) | | |
| PE | 26.4 ± 1.9^aA^ | 41.8 ± 3.5^pB^ |
| PP | 48.0 ± 3.1^bA^ | 25.0 ± 1.1^qA^ |
| PHB | 38.2 ± 2.4^bA^ | 40.2 ± 2.7^pA^ |
| PLA | 45.0 ± 2.0^bA^ | 53.2 ± 1.1^rA^ |
| *3.3.2 Soil pH, organic matter and respiration rate* | | |
| Variable: Soil pH (Table 2) | | |
| PE | 5.58 ± 0.03^bA^ | 5.67 ± 0.03^qA^ |
| PP | 5.68 ± 0.04^bA^ | 5.74 ± 0.02^qA^ |
| PHB | 5.84 ± 0.04^aA^ | 5.59 ± 0.02^pqA^ |
| PLA | 5.42 ± 0.05^cA^ | 5.45 ± 0.05^pA^ |
| Variable: Soil respiration rate (Figure 6) | | |
| PE | 5.60 ± 0.27^aA^ | 5.94 ± 0.28^pA^ |
| PP | 6.00 ± 0.25^abA^ | 6.32 ± 0.17^pA^ |
| PHB | 6.23 ± 0.12^abA^ | 6.80 ± 0.25^pA^ |
| PLA | 7.04 ± 0.30^bA^ | 6.94 ± 0.20^pA^ |

Table S3. Results of the two-way ANOVA for each response variable of the plastic leachate experiment. The data are organised following the structure of the manuscript, including a reference to corresponding tables or figures in the main manuscript. Different lower-case letters represent a significant difference between polymer treatments (Micro: a – b; Meso: p – r); different upper-case letters represent a significant difference between different sizes of the same polymer (p ≤ 0.05, ANOVA and Tukey test).

| Polymer type | Micro | Meso |
| --- | --- | --- |
| *3.1.1 Biomass and chlorophyll content of* L. perenne | | |
| Variable: Root biomass of *L. perenne* (Figure 7) | | |
| PE | 20.4 ± 2.6^aA^ | 22.8 ± 1.9^pA^ |
| PP | 25.2 ± 2.3^abA^ | 33.2 ± 1.9^qrA^ |
| PHB | 33.4 ± 2.9^bA^ | 31.6 ± 2.6^prA^ |
| PLA | 31.4 ± 1.7^bA^ | 43.4 ± 1.9^qB^ |
| Variable: Chlorophyll-b content of *L. perenne* (Figure 8) | | |
| PE | 3.96 ± 0.28^aA^ | 5.32 ± 0.32^pB^ |
| PP | 4.93 ± 0.30^abA^ | 5.22 ± 0.22^pA^ |
| PHB | 5.08 ± 0.33^abA^ | 5.31 ± 0.17^pA^ |
| PLA | 5.29 ± 0.25^bA^ | 5.28 ± 0.30^pA^ |
| *3.1.2 Soil pH, organic matter and respiration rate* | | |
| Variable: Soil pH (Table 3) | | |
| PE | 5.62 ± 0.06^aA^ | 5.66 ± 0.05^qA^ |
| PP | 5.63 ± 0.06^aA^ | 5.67 ± 0.05^qA^ |
| PHB | 5.71 ± 0.04^aA^ | 5.93 ± 0.03^pB^ |
| PLA | 5.70 ± 0.04^aA^ | 5.69 ± 0.05^qA^ |
| Variable: Soil organic matter (Table 3) | | |
| PE | 11.7 ± 0.4^bA^ | 11.4 ± 0.2^qA^ |
| PP | 12.2 ± 0.4^bA^ | 12.0 ± 0.3^qA^ |
| PHB | 14.4 ± 0.3^aA^ | 14.2 ± 0.2^pA^ |
| PLA | 13.0 ± 0.2^abA^ | 12.5 ± 0.4^qA^ |

Table S4. Estimates for number of plastic pieces in a 0.5 g treatment.

|  | Estimated number of plastic pieces | |
| --- | --- | --- |
| Polymer type | Micro | Meso |
| PE | 3490 | 246 |
| PP | 3681 | 259 |
| PHB | 2667 | 188 |
| PLA | 533 | 38 |
